# A new crystal form of a hyperthermophilic endocellulase

**DOI:** 10.1107/S2053230X14010930

**Published:** 2014-06-18

**Authors:** Misumi Kataoka, Kazuhiko Ishikawa

**Affiliations:** a Biomass Refinery Research Center, National Institute of Advanced Industrial Science and Technology (AIST), 3-11-32 Kagamiyama, Higashi-hiroshima, Hiroshima 739-0046, Japan

**Keywords:** hyperthermophile, endocellulase, *Pyrococcus furiosus*

## Abstract

The hyperthermophilic glycoside hydrolase family endocellulase 12 from the archaeon *Pyrococcus furiosus* (EGPf; Gene ID PF0854; EC 3.2.1.4) catalyzes the hydrolytic cleavage of the β-1,4-glucosidic linkage in β-glucan in lignocellulose biomass. A crystal of EGPf was previously prepared at pH 9.0 and its structure was determined at an atomic resolution of 1.07 Å. This article reports the crystallization of EGPf at the more physiologically relevant pH of 5.5. Structure determination showed that this new crystal form has the symmetry of space group *C*2. Two molecules of the enzyme are observed in the asymmetric unit. Crystal packing is weak at pH 5.5 owing to two flexible interfaces between symmetry-related molecules. Comparison of the EGPf structures obtained at pH 9.0 and pH 5.5 reveals a significant conformational difference at the active centre and in the surface loops. The interfaces in the vicinity of the flexible surface loops impact the quality of the EGPf crystal.

## Abstract 2

The hyperthermostable endocellulase from *P. furiosus* was crystallized at pH 5.5. The new crystal form has symmetry consistent with space group *C*2 and exhibits a structure different from that of the protein crystallized at pH 9.0.

## 1. Introduction   

Cellulases are the most important industrial enzymes for biomass utilization, since the enzyme plays a key role in the degradation of β-glucan cellulose. Recent research into biofuel production from lignocellulose biomass has accelerated the development of cellulases optimized for efficient biomass breakdown to monosaccharides, known as saccharification. A hyperthermophilic cellulase would be very useful in industrial applications because enzymatic reactions occurring at high temperature have many merits, such as a reduced risk of microbial contamination, increased solubility of the substrate and improved transfer rate. Therefore, much research has focused on developing thermophilic cellulases with high activity.

Hyperthermophilic β-1,4-endocellulases (endo-type cellulases) have been found in the genome databases of several hyperthermophilic archaea. The hyperthermophilic archaea *Pyrococcus horikoshii* and *P. furiosus* have glycoside hydrolase (GH) family 5 (EGPh) and family 12 endocellulases (EGPf), respectively. Each family of enzymes shows different substrate specificities and exhibits hydrolytic activity at high temperatures. The optimal and denaturing temperatures of EGPh are 100 and 103°C, respectively (Kim & Ishikawa, 2013 ▶), and those of EGPf are 100 and 112°C, respectively (Bauer *et al.*, 1999 ▶). The crystal structures of two hyperthermophilic endocellulases have been determined (Kim & Ishikawa, 2010 ▶; Kim *et al.*, 2012 ▶). The structure of EGPf was determined at an atomic resolution of 1.07 Å (Kim *et al.*, 2012 ▶; PDB entry 3vgi). Under the crystallization conditions used by Kim and coworkers, the EGPf crystal form has symmetry consistent with space group *P*2_1_2_1_2 and one EGPf molecule is present per asymmetric unit. The optimum pH of EGPf is reported to be approximately pH 6.0 (Bauer *et al.*, 1999 ▶), but the crystal was prepared at pH 9.0 (Kataoka *et al.*, 2012 ▶). Here, we describe a new crystal form of EGPf prepared at pH 5.5 and compare it with the previously solved EGPf structure.

## 2. Materials and methods   

### 2.1. Protein preparation   

We prepared recombinant EGPf using a method similar to that described previously (Kim *et al.*, 2012 ▶). The plasmid containing the full-length EGPf used the vector pET-11a (Novagen, Madison, Wisconsin, USA) and was introduced into *Escherichia coli* strain BL21 (DE3) pLysS. The truncated protein gene (EGPfΔN30), with a deletion of 30 amino-acid residues (signal sequence and the proline and hydroxyl residue-rich regions) at the N-terminal region of EGPf, was constructed by PCR and inserted into the pET-11a vector. pET-11a was introduced into *E. coli* BL21 (DE3) cells for recombinant protein expression. The cells were cultured in LB containing 100 mg l^−1^ sodium ampicillin at 37°C to an OD_600_ of 0.6, and isopropyl β-d-1-thiogalactopyranoside (IPTG) was then added. After 16 h culture at 30°C, the cells were harvested by centrifugation (5000*g*, 15 min, 4°C). The cells were suspended in 20 m*M* Tris–HCl pH 8.0 containing 0.7 *M* ammonium sulfate and then homogenized by ultrasonication (40 W, 20 kHz) for 15 min on ice. The homogenates were heated at 70°C for 15 min. 

After removing the cell debris by centrifugation (15 000*g*, 20 min, 4°C), the enzyme solution was filtered (0.2 µm) and applied to hydrophobic interaction column chromatography (using a HiTrap Phenyl column). The flow rate for the column chromatography was 2.0 ml min^−1^ using 20 m*M* Tris–HCl pH 8.0 buffer with a gradient of 0.7–0 *M* ammonium sulfate. Gel-filtration chromatography (using a HiLoad 26/60 Superdex 200 pg column) was carried out using 20 m*M* Tris–HCl pH 8.0 buffer with a flow rate of 2.0 ml min^−1^. The purity and molecular weight of the protein were analyzed by SDS–PAGE. The concentration of EGPfΔN30 (molecular weight 30 540.48 Da) was determined from the UV absorbance at 280 nm, using 81 790 as the molar extinction coefficient, which was calculated from the protein sequence (UniProt ID E7FHY8; Gill & von Hippel, 1989 ▶).

### 2.2. Crystallization   

The purified EGPfΔN30 was concentrated to 17 mg ml^−1^ and then dialyzed against 20 m*M* Tris–HCl pH 8.0 using an Amicon Centricon YM-10 (Millipore, Billerica, Massachusetts, USA) by centrifugation (5000*g*, 4°C). The crystals of EGPfΔN30 were grown at 22°C using a reservoir solution composed of 100 m*M* CHC (2:3:4 citric acid:HEPES:CHES) buffer pH 5.5, 200 m*M* lithium sulfate, 5%(*v*/*v*) ethanol by the hanging-drop vapour-diffusion method. Typically, drops consisting of 1 µl protein solution and 1 µl reservoir solution were equilibrated against 450 µl reservoir solution.

### 2.3. Data collection and processing   

The selected crystals were harvested and immersed in cryoprotectant solution consisting of 30%(*v*/*v*) glycerol in mother liquor. The soaked crystal was collected using a Cryo-Loop (Hampton Research, Aliso Viejo, California, USA) and immediately flash-cooled under a stream of nitrogen gas at −173°C. X-ray diffraction data for a single crystal measurement were collected using an MX-300HE CCD detector (Rayonix, Evanston, Illinois, USA) on the SPring-8 BL44XU beamline (Hyogo, Japan). The diffraction data set extended to 1.68 Å resolution and was collected at a wavelength of 0.9 Å. The crystal-to-detector distance was 220 mm. The crystal was rotated 180° with an oscillation angle of 0.5° per frame. The data collected from diffraction measurements were merged, indexed, integrated and scaled using the programs in the *HKL*-2000 software package (Otwinowski & Minor, 1997 ▶). Data-collection statistics are presented in Table 1 ▶.

### 2.4. Structure determination and refinement   

The EGPfΔN30 structure was determined by molecular replacement with *MOLREP* (Vagin & Teplyakov, 2010 ▶) in the *CCP*4 package (Winn *et al.*, 2011 ▶), using the structure of EGPfΔN30 in the *P*2_1_2_1_2 form as a search model (Kim *et al.*, 2012 ▶; PDB entry 3vgi). Structure model building was performed with *Coot* (Emsley *et al.*, 2010 ▶). The structure was refined using *REFMAC*5 (Murshudov *et al.*, 2011 ▶).Water molecules were introduced at peaks over 3 r.m.s.d. in the *F*_o_ − *F*_c_ difference Fourier map fulfilling reasonable interactions with the protein model. A Ramachandran plot of the final structure was validated using *PROCHECK* (Laskowski *et al.*, 1993 ▶). The values of the r.m.s.d. for comparisons of structures were calculated using *SUPERPOSE* (Krissinel & Henrick, 2004 ▶). Figures were prepared using *PyMOL* (DeLano, 2002 ▶). The refinement statistics are presented in Table 1 ▶. The interface between EGPfΔN30 and symmetric molecules was calculated using *PISA* (*Protein Interfaces, Surfaces and Assemblies*; Krissinel & Henrick, 2007 ▶).

## 3. Results and discussion   

### 3.1. Structure of EGPf at pH 5.5   

**Figure 1:**
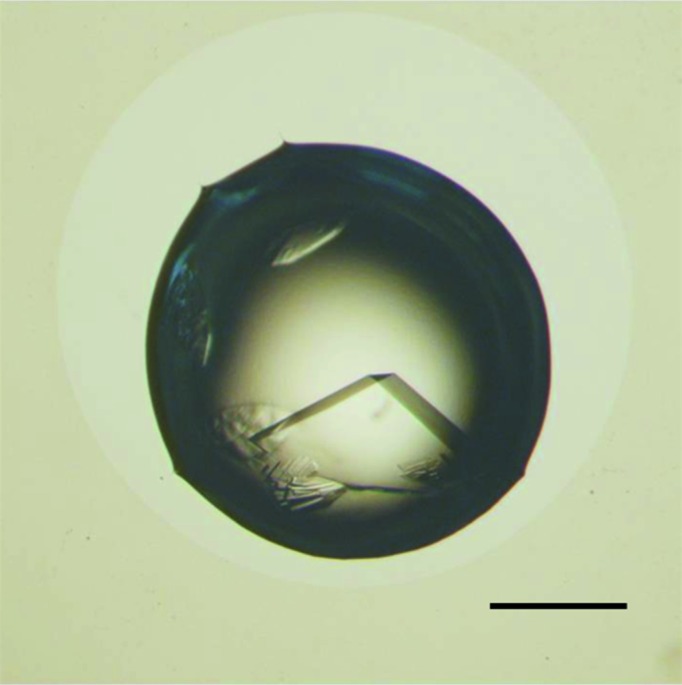
A photograph of the EGPfΔN30 crystals prepared at pH 5.5. The scale bar corresponds to 0.5 mm.

**Figure 2:**
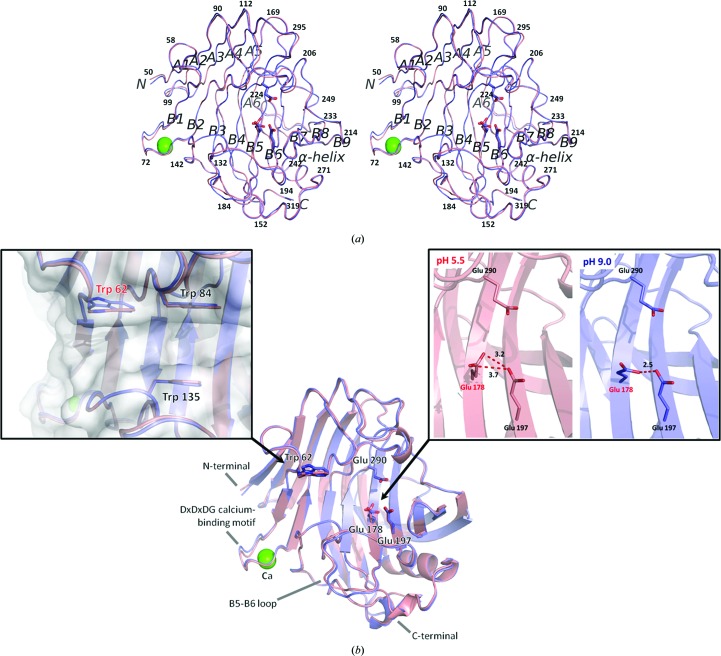

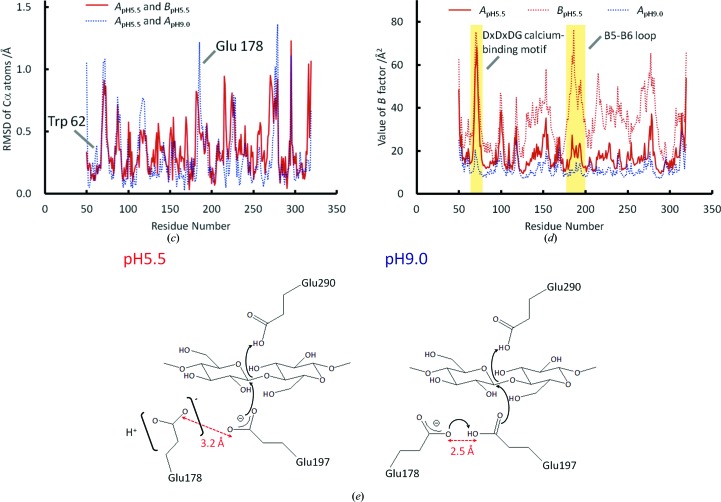
Comparison of the EGPfΔN30 structure at pH 5.5 and at pH 9.0 identified by colour: red, pH 5.5; blue, pH 9.0. (*a*) Wall-eyed stereoview of the overall crystal structure of EGPfΔN30 drawn as a ribbon model viewed from the front. The two EGPfΔN30 structures are superimposed on each other. (*b*) The structure of the entrance to the active-site cleft is changed between *A*_pH5.5_ and *A*_pH9.0_, as are the structures of the catalytic residues. (*c*) The r.m.s.d. values of the C^α^ atoms of *A*_pH5.5_/*B*_pH5.5_ and *A*_pH5.5_/*A*_pH9.0_. (*d*) *B* factors of the amino-acid residues of EGPfΔN30 at pH 5.5 (*A*_pH5.5_ and *B*_pH5.5_) and pH 9.0 (*A*_pH9.0_). Hydrogen bonds between the two molecules are indicated by a dotted red line. (*e*) Catalytic mechanism of EGPf in the first half-reaction. Here, the typical of a retaining enzyme is depicted in a schematic diagram. The two glucose residues correspond to the productive binding mode. pH 9.0: the acidic side chain of Glu178 adjacent to the nucleophile Glu197 maintains a negative charge. pH 5.5: the nucleophile Glu197 maintains a negative charge without the side chain of Glu178.

EGPf appears to be a secretory enzyme because of its signal sequence at the N-terminus. Recombinant EGPf without the signal sequence was expressed using the pET system. No recombinant enzyme crystals were obtained using the Crystal Screen (Hampton Research, Aliso Viejo, Califonia, USA) or Wizard I and II (Emerald Bio, Bainbridge Island, Washington, USA) crystallization screening kits. However, the recombinant product of a truncated protein gene (EGPfΔN30), in which the N-terminal 30 amino-acid residues (signal sequence and the proline and hydroxyl residue-rich regions) were deleted from EGPf, was crystallized at pH 9.0, and the structure was determined at 1.07 Å resolution (Kim *et al.*, 2012 ▶; PDB entry 3vgi). Under these crystallization conditions, crystals grew with symmetry consistent with space group *P*2_1_2_1_2, and one EGPf molecule is present per asymmetric unit. However, the enzymatic optimum pH of EGPf was reported to be approximately pH 6.0 (Bauer *et al.*, 1999 ▶). Therefore, we attempted to prepare crystals at the more physiologically relevant pH of 5.0–6.0 in order to obtain the structure of the active site in EGPf at these conditions. Based on initial screening results, high-quality crystals of EGPfΔN30 were obtained using a reservoir solution consisting of 100 m*M* CHC (2:3:4 citric acid:HEPES:CHES) buffer pH 5.5, 200 m*M* lithium sulfate, 5%(*v*/*v*) ethanol at 22°C. The average size of each crystal was about 0.7 × 0.5 × 0.3 mm after one week (Fig. 1 ▶). This was a new crystal form with symmetry consistent with space group *C*2 that diffracted to 1.68 Å resolution. The data-collection statistics are summarized in Table 1 ▶. Determination of the structure of EGPfΔN30 was carried out by the molecular-replacement method using the previous structural data (Kim *et al.*, 2012 ▶; PDB entry 3vgi). Two molecules of EGPfΔN30, labelled *A*_pH5.5_ and *B*_pH5.5_, were identified in the crystallographic asymmetric unit. The final model contains two monomer molecules with 270 amino-acid residues each. After refinement, the *R* factors were estimated to be *R*_work_ = 0.181 and *R*_free_ = 0.217. The structure of the enzyme consists of a β-jelly-roll fold (Fig. 2 ▶ *a*).

### 3.2. Comparison to the previously determined EGPf structure   

The r.m.s.d. of the C^α^ atoms between *A*_pH5.5_ and *B*_pH5.5_ was 0.3 Å (Fig. 2 ▶ *b*). In both molecules, the D*x*D*x*DG calcium-binding motif (Asp68–Glu76 and Asp142) was present in the loop region between the B1 and B2 strands and exhibited high *B*-factor values (Fig. 2 ▶ *c*). In *B*_pH5.5_, poor electron density was observed for the loop regions of B3–A5 (Gly131–Asp156), B5–B6 (Thr182–Asp194) and α-helix–B4 (Ser272–Glu283), but the regions were interpretable. On the other hand, EGPf crystallized at pH 9.0 has symmetry consistent with *P*2_1_2_1_2, with unit-cell parameters *a* = 58.0, *b* = 118.7, *c* = 46.8 Å (Kim *et al.*, 2012 ▶; PDB entry 3vgi). Under the previous crystallization conditions, one molecule exists in the asymmetric unit (labelled *A*_pH9.0_). The r.m.s.d. of the C^α^ atoms between structures *A*_pH5.5_ and *A*_pH9.0_ was 0.2 Å (Fig. 2 ▶ *b*). Comparison of the structures at pH 5.5 and 9.0 showed that the structures of both main chains are the same. However, conformational changes were observed in the side chains of Trp62 and Glu178 (Fig. 2 ▶ *a*) located at the active-site cleft. Trp62 seems to contribute to the substrate binding (Kim *et al.*, 2012 ▶; PDB entry 3vgi). The torsion angle (χ_2_) of the indole ring of Trp62 differs by approximately 20° between *A*_pH5.5_ and *A*_pH9.0_. Trp62 is not particularly mobile as the *B* factor of Trp62 is ∼20 Å^2^. Trp62 is located at subsite −4, at the entrance to the nonreducing side of the active-site cleft. On the other hand, the dihedral angle of the carboxyl group of Glu178 between *A*_pH5.5_ and *A*_pH9.0_ is approximately 80°. Because of this, the distance between Glu178 OE1 and Glu197 OE2 in *A*_pH5.5_ (3.2 Å) is larger than that in *A*_pH9.0_ (2.5 Å) by 0.7 Å. Glu178 is located to the back of two catalytic residues (Glu197, nucleophile; and Glu290, proton donor) and it is thought to be the proton donor to Glu197 (cellulase 12A from *Thermotoga maritima*; Cheng *et al.*, 2011 ▶). Although the *B*-factor values for Glu197 of *A*_pH5.5_ and *B*_pH5.5_ are 16 and 35 Å^2^, respectively, the conformation of Glu178 did not change between the apo forms (*A*_pH5.5_ and *B*_pH5.5_). No other significant differences at the active centre were observed among the *A*_pH9.0_, *A*_pH5.5_ and *B*_pH5.5_ molecules. This result suggests that the conformational change of Glu178 is due to protonation of the carboxyl group of Glu178 and/or Glu197 at acidic pH. The position or state of nucleophile Glu197 is stabilized by Glu178 at pH 9.0. Glu178 seems to play a role in controlling the optimum pH of the enzymatic activity. From the catalytic mechanism of cellulase 12A from *T. maritima* (Cheng *et al.*, 2011 ▶), Glu197 is identified as the catalytic nucleophile of EGPf (Fig. 2 ▶ *e*). In the first half-reaction, the acidic side chain of Glu178 adjacent to the nucleophile Glu197 is believed to maintain a negative charge (Fig. 2 ▶ *e*), as suggested by Cheng *et al.* (2011 ▶). This catalytic mechanism was supported by the structural data at pH 9.0 (Fig. 2 ▶ *b*). However, the structural data at pH 5.5 (Fig. 2 ▶ *b*) suggest another catalytic mechanism (Fig. 2 ▶ *e*). It is speculated that the side chain of Glu178 located at a distance of 3.2–3.7 Å from the nucleophile Glu197 is protonated and the nucleophile Glu197 maintains a negative charge in the first half-reaction.

### 3.3. Interfaces with symmetry-related molecules   

**Figure 3:**
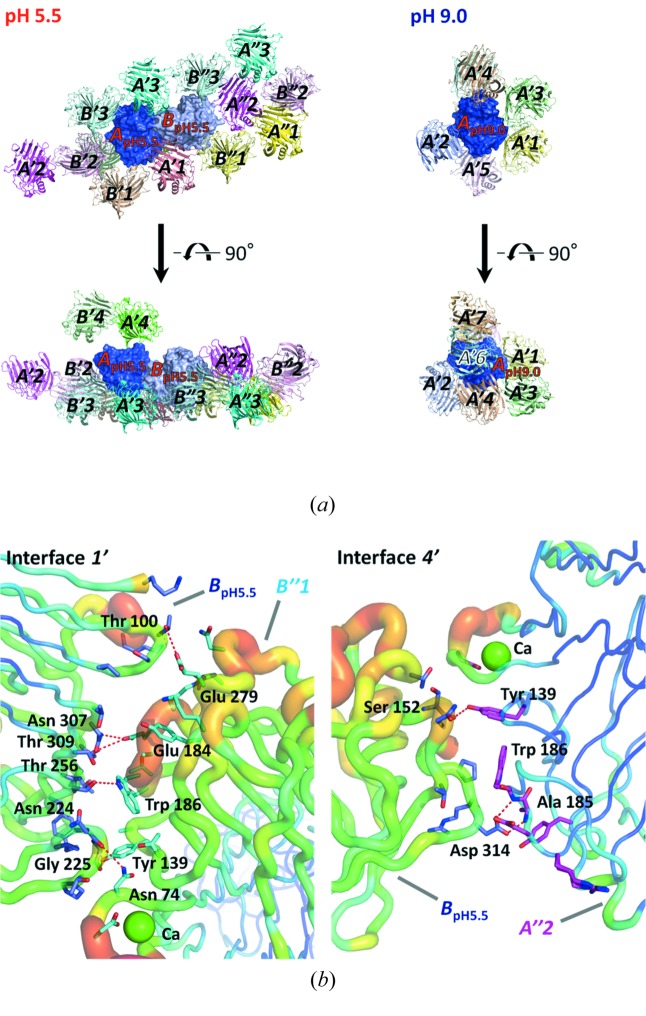
Symmetry-related molecules and interfaces of *A*_pH5.5_ and *A*_pH9.0_. The single primes (′) refer to molecules that are in the first layer relative to *A*_pH5.5_ or *A*_pH9.0_ and the double primes (′′) refer to molecules that are in a second layer relative to *B*_pH5.5_. (*a*) Seven symmetry-related molecules, drawn as cartoon models, are viewed from the front and upper side. Characters in the molecules correspond to the interaction molecules in Table 2 ▶. (*b*) Interfaces 1′ and 4′ are drawn as tube models. Rainbow colours are used to show the high (red) and low (blue) *B* factors of the amino-acid residues.

The EGPfΔN30 structures at pH 5.5 and at pH 9.0 each interact with seven symmetry-related molecules (Fig. 3 ▶ *a*, Table 2 ▶): single primes (′) for the interacting molecules refer to molecules that are in the first layer relative to a central molecule (*A*_pH5.5_ or *A*_pH9.0_) and double primes (′′) refer to molecules that are in a second layer relative to the central molecule (*B*_pH5.5_). That is, seven interfaces (1–3, 1′–4′) at pH 5.5 and four interfaces (1′′–4′′) at pH 9.0 were formed (Table 2 ▶). The interfaces between monomers of the central molecule and the symmetry-related molecules are summarized in Table 2 ▶. The *B*-factor values of the amino-acid residues of the two determined structures are shown in Fig. 2 ▶(*c*). The average values for *A*_pH5.5_, *B*_pH5.5_ and *A*_pH9.0_ are 18, 33 and 12 Å^2^, respectively. In *B*_pH5.5_, the *B* factors of the overall and loop region of the surface are higher than for *A*_pH5.5_ and *A*_pH9.0_. In particular, the *B* factors of the D*x*D*x*DG calcium-binding motif and the B5–B6 loop region in *A*_pH5.5_/*B*_pH5.5_ exhibit higher values (44/54 Å^2^ and 19/51 Å^2^) than in *A*_pH9.0_ (14 and 11 Å^2^) (Figs. 2 ▶ *c* and 3 ▶ *b*). In *B*_pH5.5_, two interactions, 1′ (*B*_pH5.5_–*B*′′1) and 4′ (*B*–*A*′′2), in these regions have higher *B*-factor values than the other five interactions (1, 2, 3, 2′ and 3′) (Table 2 ▶). These interactions are likely to weaken the molecular packing because of the high fluctuation and flexibility of these regions. In contrast to the EGPfΔN30 structures at pH 5.5, *A*_pH9.0_ has stronger packing because of the lower flexibility of the interfaces. In conclusion, crystal packing is weaker and the quality of the EGPfΔN30 crystal at pH 5.5 is lower than at pH 9.0 because of the flexible interfaces 1′ and 4′.

## Supplementary Material

PDB reference: hyperthermophilic endocellulase, 3wq7

## Figures and Tables

**Table 1:** Data-collection and refinement statistics for the structure of EGPfN30 at pH 5.5

Data collection	
Wavelength ()	0.9
Space group	*C*2
Unit-cell parameters (, )	*a* = 134.7, *b* = 62.6, *c* = 86.3, = 95.1
Molecules per asymmetric unit	2
Matthews coefficient (^3^Da^1^) (Matthews, 1968 ▶)	2.5
Solvent content (%)	51
Resolution range ()	50.01.68 (1.711.68)
Total No. of observed reflections	311544 (15000)
No. of unique reflections	81623 (4029)
Average *I*/(*I*)	14.9 (3.7)
*R*_merge_ †	0.068 (0.367)
Multiplicity	3.8 (3.8)
Completeness (%)	99.9 (99.9)
Refinement	
No. of atoms	
Protein	4390
Glycerol	102
Ca^2+^	4
Water	468
Resolution used in refinement ()	43.01.68
*R*_work_ ‡/*R*_free_ §	0.181/0.217
Wilson *B* factor (^2^)	18
R.m.s.d., bond distances¶ ()	0.03
R.m.s.d., bond angles ¶ ()	2.5
Mean overall *B* factor (^2^)	27
Ramachandran plot	
Most favoured regions (%)	96.6
Disallowed regions (%)	0.0
PDB code	3wq7

† *R*_merge_ = 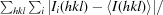 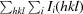, where *I_i_*(*hkl*) is the intensity of the *i*th measurement of reflection *hkl*, including symmetry-related reflections, and *I*(*hkl*) is their average.

‡ *R*_work_ = 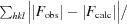 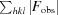.

§ *R*_free_ is *R*_work_ for approximately 5% of the reflections that were excluded from the refinement.

¶ R.m.s.d. bond distances and angles are r.m.s.d.s from ideal values (Engh Huber, 1991 ▶).

**Table 2:** Interfaces between monomers of the determined molecule and the symmetric molecules In the interface, single primes () refer to interactions with central molecule (*A*_pH5.5_ or *A*_pH9.0_) and double primes () refer to interactions with central molecule (*B*_pH5.5_). In the interaction molecule, single primes () of the interaction molecules refer to molecules that are in the first layer relative to a central molecule (*A*_pH5.5_ or *A*_pH9.0_) and double primes () refer to molecules that are in a second layer relative to that central molecule (*B*_pH5.5_).

Interface	Interacting molecule	Symmetry operation	Interface area (^2^)	Solvation free energy gain (^i^ *G*)† (kcalmol^1^)	Hydrogen bonds	Salt bridges	*B* factor‡ (^2^)
*A*_pH5.5_							
1	*A*1	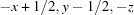	410	0.5	6	0	16/16
2	*A*2	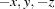	360	6.1	4	0	17/17
3	*B*1	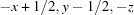	200	1.9	1	0	27/25
*B*_pH5.5_							
1	*B*1	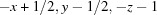	410	2.1	6	0	38/52
2	*A*_pH5.5_	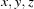	380	2.0	6	0	17/15
3	*A*1	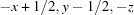	310	1.8	3	3	19/18
4	*A*2	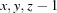	220	0.8	3	0	42/27
*A*_pH9.0_							
1	*A*1	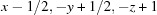	500	2.3	6	0	12/13
2	*A*2	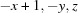	480	1.3	14	2	12/12
3	*A*3	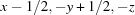	220	0.5	1	0	14/13
4	*A*4	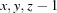	200	3.5	0	0	13/16

† The sum values of the gain on complex formation for the two surfaces.

‡ Value of *B* factor at the interface belonging to each monomer.
